# Inappropriate Journal Authorship, Disputes, Plagiarism, and Mistrust in the Institution: Different Beasts ... Same Problem

**DOI:** 10.5041/RMMJ.10514

**Published:** 2023-10-29

**Authors:** Itamar Ashkenazi, Oded Olsha

**Affiliations:** 1 The Ruth and Bruce Rappaport Faculty of Medicine, Technion–Israel Institute of Technology, Haifa, Israel; 2 General Surgery Department, Rambam Health Care Campus, Haifa, Israel; 3 General Surgery Department [Emeritus], Shaare Zedek Medical Center, Jerusalem, Israel; 4 Hadassah Faculty of Medicine [Emeritus], Hebrew University of Jerusalem, Jerusalem, Israel

**Keywords:** Author dispute, authorship criteria, ghost authorship, gift authorship, plagiarism, publication ethics

**To the Editor**:

We would like to thank Professor Marshall Lichtman for his letter, his interesting proposal, and using this venue to promote discussion of the topic. Professor Lichtman proposed a numerical calculation for authorship based on the authors’ perceptions of their relative contribution to a scientific publication,^1^ an idea also suggested by Jozsef Kovacs.^2^ The only limitation imposed by this system is that the total of all authors’ fractional contributions to any one publication equals no more than one. Lichtman’s interesting proposal serves as a disincentive to offer gift authorship to colleagues whose contributions were minimal, if they contributed at all.

However, we question whether this proposal will solve authorship problems in academic publishing. Take, for example, coercive authorship. If Lichtman’s proposal is adopted, certain individuals’ demands might not be satisfied by being given a place in the authors’ byline. These individuals would now demand a substantial “share” within the fractional contribution to legitimize their inclusion. In such a case, the relative “contribution” of legitimate authors can only decrease. Such a scenario could potentially transform legitimate contributors into ghost authors, further exacerbating the problem of misattributed authorship.

Contrary to Lichtman’s model, in which the only parameter examined is the authors’ roles in deciding who contributed and the degree to which they contributed, our study evaluated the impact of the institutions with which the authors were affiliated on authorship misconduct. We evaluated authorship dispute since it is a commonly encountered situation. We maintain that the issues underlying these disputes are much larger than just a personal disagreement between individual contributors. Typically, although several parties may be involved in a dispute (disputing authors and their institution(s)), only the disputing authors are officially engaged in the conflict; the institutions become officially involved only if it is reported to them. Lichtman’s model, however, assumes that the institutions of disputing authors have no influence on the evolution of the conflict. The results of our study question Lichtman’s assumption. We showed that increasing experience with authorship misconduct is associated with lower Trust Scores in the respondents’ institutions. In fact, most respondents preferred not to involve their administration in resolving a conflict if there was an authorship dispute. These respondents did not perceive their institutions to be passive or neutral third parties.

An idealistic view of academia leads to shock at the thought that a scientist, who has spent years studying, exploring, and teaching, would be willing to engage in misconduct such as placing one’s name on a scientific publication without justification or, worse, falsifying and fabricating experimental results. The incentive is the pressure to publish at any cost. This pressure is not inborn. Rather, this pressure is the consequence of institutional demands.

Many organizations, such as the US Office of Research Integrity, argue that authorship complaints should be tagged as authorship disputes rather than acts of misconduct such as plagiarism.^3^,^4^ While plagiarism is considered one of the worst offenses, the term “authorship dispute” understates serious underlying problems. The term “authorship dispute” allows offenders and relevant institutions to disregard possible misconduct. We argue otherwise. In most cases, a direct link will be found between author disputes and authorship misconduct, including plagiarism.

Take, for example, author displacement. According to The European Code of Conduct for Research Integrity, “Plagiarism is using other people’s work or ideas without giving proper credit to the original source.”^5^ Thus, in an academic system that highly values author placement order within the list of authors, having an individual in an “inferior” position that does not correspond to their actual contribution is a *discredit* and constitutes plagiarism.

**Figure 1:**
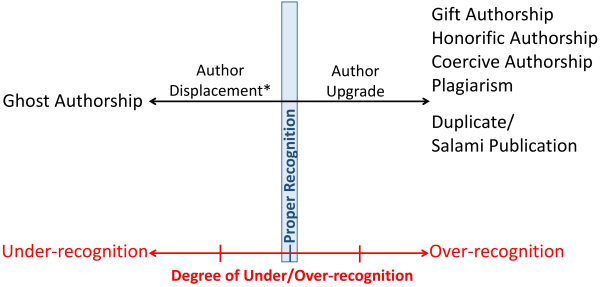
The Spectrum of Inappropriate Crediting of Individual Contributions to Scientific Publications. *The degree of under-recognition is determined by the degree of displacement.

Both plagiarism and other causes commonly underlying authorship disputes belong within the spectrum of inappropriate crediting of an individual’s contributions to the scientific record (Figure 1). Ghost authorship is at one extreme, while gift/honorary/coercive authorship, duplicate publications, and plagiarism are on the other extreme of this spectrum. Author displacement lies between proper recognition and ghost authorship. Plagiarism, ghost authorship, and author displacement are based on a worldview that does not respect proper recognition, in which one or more individuals are over-recognized while others are under-recognized, or not recognized at all. Plagiarism cannot exist without ghost authorship or author displacement. There is always a victim on the other side of the spectrum.

We believe that the scientific establishment must stop diminishing the significance of authorship disputes. The root causes of such disputes and the system failures to resolve them should be sought and addressed. If underlying misconduct is encountered, institutions must not ignore it, regardless of the seniority or tenure of the faculty member involved. We wish to quote one of our survey respondents who commented on the importance of the active role of institutions: “Within our institute, we have clear rules and guidance on how authorship is handled, and we rarely have issues with authorship. Therefore, in our environment, authors can handle this [dispute] themselves.” Our study clearly showed that Trust was higher and that gift authorship rates were lower in institutions that made known their authorship policies.

Institutions should actively foster a culture embracing research and publication integrity. To that end, institutions should seek to be more proactive in the prevention and resolution of authorship disputes. Integral steps include establishing and publishing a non-ambiguous policy, examination of the source of such disputes, and setting in motion processes that will promote equitable authorship dispute resolution.

We would like to thank Professor Lichtman for raising the issue of proper attribution of authorship, which is the basis for avoiding authorship disputes.
